# Dental implant surface morphology, chemical composition, and topography following double wavelength (2780/940 nm) laser irradiation. An in vitro study

**DOI:** 10.1002/cre2.709

**Published:** 2023-01-01

**Authors:** Peter Fahlstedt, Ann Wennerberg, Dagmar F. Bunæs, Stein A. Lie, Knut N. Leknes

**Affiliations:** ^1^ Department of Clinical Dentistry, Faculty of Medicine University of Bergen Bergen Norway; ^2^ Department of Prosthodontics, Institute of Odontology, Sahlgrenska Academy University of Gothenburg Gothenburg Sweden

**Keywords:** alteration, dental implant, laser, titanium

## Abstract

**Objective:**

The aim of this in vitro study was to evaluate morphology alterations, chemical composition, and topography of moderately rough dental implants following double‐wavelength laser irradiation.

**Material and Methods:**

Commercial‐grade titanium dental implants representing different surface characteristics (Osseospeed [OS], TiUnite [TiU], and Roxolid SLActive [RS]) were used. Laser irradiation was performed using a computer‐controlled robotic device with calibrated energy/power settings and deionized water spray. Micro‐, nano‐morphology surface alterations, chemical composition, and surface topography (*S*_a_, *S*_ds_, *S*_dr_) in the test group (laser plus water), control group A (water only), and control group B (no treatment) were analyzed using scanning electron microscopy (SEM), energy‐dispersive X‐ray analysis (EDX), and white light laser profilometer (Interferometry).

**Results:**

SEM‐evaluation revealed minor between‐group differences in micro‐ and nano‐morphology within each implant system. Significant overall differences in surface element content were observed between the test and control group B for all implant systems (*p* < .05). For the test compared with control group B, statistically significantly higher oxygen content was detected for OS and RS (*p* < .05), a corresponding significant difference was detected for carbon for TiU (*p* < .05). For RS, a significantly lower content of titanium and zirconium was detected within the test group (*p* < .05). A significant difference in topography between test and control group B was observed for OS (*S*_a_: *p* = .039 and *S*_dr_: *p* = .041) with the highest roughness value for control group B.

**Conclusions:**

Altered chemical composition and surface topography were observed for all implant surfaces compared with untreated control following double wavelength laser irradiation. A clinical evaluation of the impact of the altered surface composition following double wavelength laser irradiation on the ability to reosseointegrate appears warranted.

## 1 INTRODUCTION

Free‐running pulsed Er:YAG and Er,Cr:YSGG lasers (erbium lasers) have been shown to yield efficient removal of artificial biofilm at titanium surfaces in vitro (Alagl et al., 2019; Bolhari et al., 2014; Larsen et al., 2017; S. H. Park et al., 2020; Taniguchi et al., 2013) as well as calculus on explanted dental titanium implants (Gholami et al., 2018; Secgin‐Atar et al., 2021; Takagi et al., 2018). The decontamination and interaction with the titanium implant surface micro‐texture have been reported directly proportional to applied fluence (energy/area) (Chegeni et al., 2020; Ercan et al., 2014; Miranda et al., 2015).

The bactericidal effect of diode laser irradiation on contaminated titanium surfaces correlates with applied intensity (power/area) has been shown but without observations of surface topography alterations (Valente et al., 2017; Wawrzyk et al., 2021). Further, diode laser irradiation under water spray has been shown not to cause adverse thermal effects regardless of power setting (Castro et al., 2007; Fahlstedt et al., 2020). In fact, recent in vitro studies evaluating titanium implant body temperature following dual wavelength (Er,Cr:YSGG 2780 nm/diode 940 nm) laser irradiation have demonstrated temperatures close to applied water‐spray temperature (20–24°C) (Fahlstedt et al., 2020; Haidary et al., 2019) without affecting of implant microstructure.

The ability of implant titanium surfaces to osseointegrate appears correlated to the topographical microroughness and may also be linked to nano‐roughness, chemical composition, and physical properties (Albrektsson & Wennerberg, 2019). Any surface microstructure modification and roughness may alter the surface nano‐texture and chemistry (Albrektsson & Wennerberg, 2004). None of the decontamination approaches of 14 different techniques evaluated in vitro for the cytocompatibility of contaminated titanium surfaces succeeded in restoring the pristine surface texture (Jin et al., 2019). Following laser decontamination, evaluating human cell response in vitro on previously contaminated titanium surfaces, studies have presented divergent outcomes depending on laser wavelength, energy applied, and type of implant surface (Ayobian‐Markazi et al., 2015; Chellini et al., 2017; Schwarz, 2006). Following irradiation, adverse effects on the titanium implant surface may occur. Heating may produce surface cracking, melting or ablation potentially affecting implant bioclinical properties including reosseointegration (Ercan et al., 2014; Fahlstedt et al., 2020; Secgin‐Atar et al., 2021). Studies on surface breakdown thresholds following pulsed laser suggest an implant system‐specific interaction between laser energy and implant surface composition (Fahlstedt et al., 2020; J. H. Park et al., 2012). An increase in implant body temperature above a 47°C threshold must be avoided (Eriksson & Albrektsson, 1983, 1984) to reduce the risk of jeopardizing bone vitality accentuated by the high thermal conductivity of titanium (Matys et al., 2016).

Scanning electron microscopy (SEM), commonly used for qualitative evaluation of micro‐ and nano‐texture morphology of the titanium surface, has been employed to evaluate possible alterations following different decontamination techniques (Chegeni et al., 2020; Hakki et al., 2017; Schwarz, 2006; Takagi et al., 2018). However, for an accurate three‐dimensional (3D) characterization of quantitative surface roughness, techniques that supply spatial and hybrid parameters (*S*_a,_ *S*_ds_, *S*_dr_) are required (Cao et al., 2018; J. H. Park et al., 2012; Wennerberg & Albrektsson, 2000). To analyze the semi‐quantitative chemical and elemental composition of titanium surfaces, an energy‐dispersive X‐ray is well‐accepted (Nejem Wakim et al., 2018; Souza et al., 2014; Dias et al., 2020; Yao et al., 2020).

Literature reviews have failed to consent to a safe and preferred protocol for laser decontamination and how to disinfect contaminated dental implants, likely due to study design heterogeneity and lack of precisely defined laser specifications and parameters (Kamel et al., 2014; Smeo et al., 2018). Several studies investigating different parameters such as wavelength, pulse length, repetition rate of pulses, measured output fluence and intensity, light deliverance system, applied water irrigation, and time of irradiation have demonstrated an urgent need for transparent, optimized, and standardized laboratory set‐up evaluation before clinical application (Fahlstedt et al., 2020; Takagi et al., 2018; Tunér & Jenkins, 2016). Alterations in the surface morphology, chemical composition, and topography following double wavelength laser irradiation have, to our knowledge, not been reported for titanium dental implant surfaces. This study evaluates potential alterations in the qualitative surface micro‐and nano‐texture morphology, the chemical composition, and the quantitative and qualitative surface roughness (*S*_a_, *S*_ds_, *S*_dr_), of different implant systems using a validated in vitro protocol for double wavelength laser irradiation. We hypothesized that the micro‐, nano‐morphology, chemical composition, and the surface roughness would be altered for irradiated implant surfaces compared with nonirradiated surfaces.

## 2 MATERIAL AND METHODS

### 2.1 Dental implants

**Figure 1:**
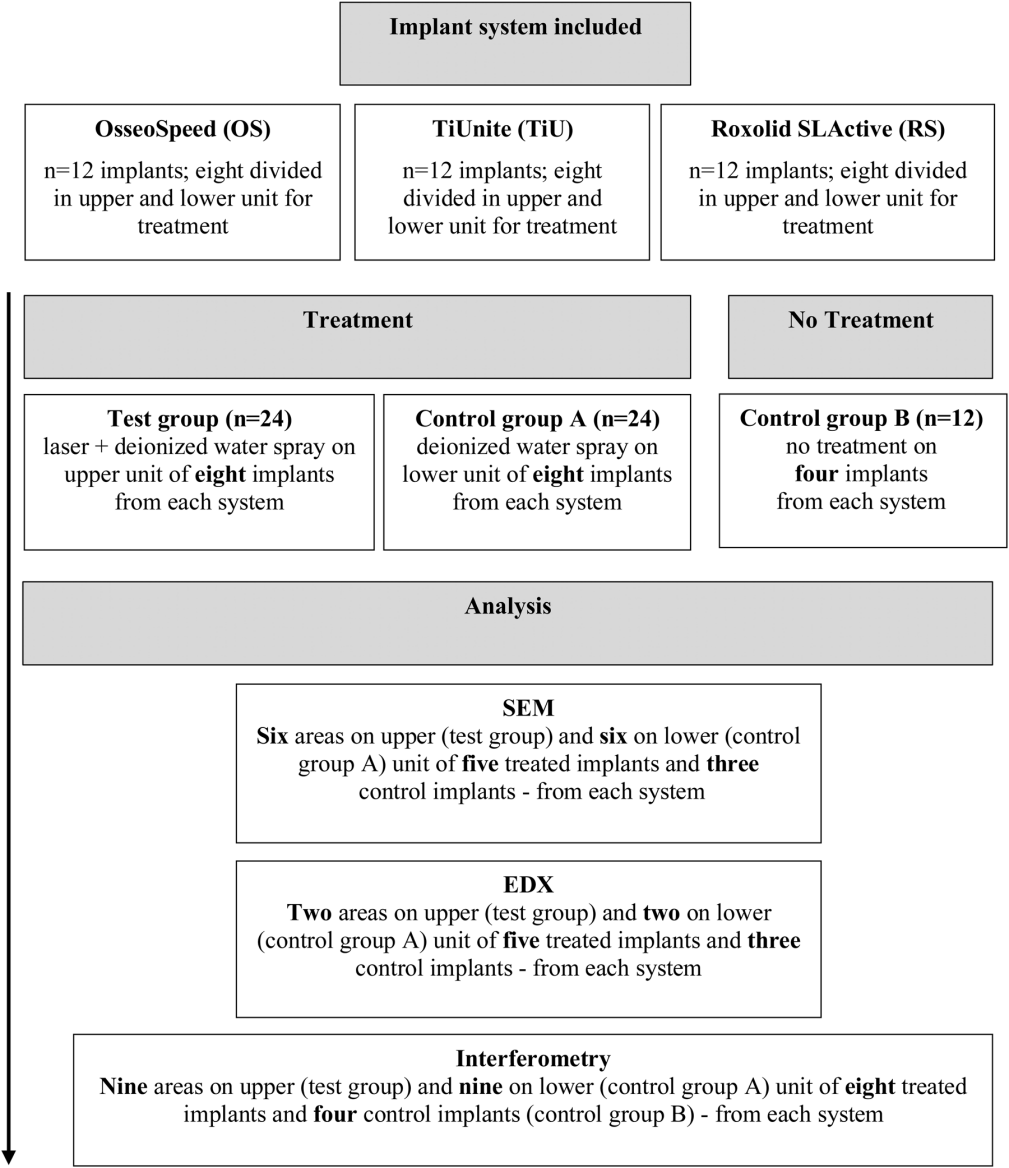
Study design. In total, 36 titanium dental implants representing three types of treatment and analyses. Before treatment, all implants were randomly allocated into the test or control group. Methods of analyses were scanning electron microscopy (SEM): evaluation of qualitative surface micro‐, nano‐texture morphology; energy‐dispersive X‐ray (EDX): analysis of chemical composition; interferometry (Light Profilometer): qualitative 3D evaluation and quantitative surface roughness, *S*_a_, *S*_ds_, *S*_dr_. Same implants were used for SEM, EDX, and interferometry. For interferometry, additionally, three implants in test group and control group A were used. 3D, three‐dimensional.

Thirty‐six new titanium dental implants, 12 each from three principal implant systems representing three different surface modifications (Table 1), were used in this in vitro study. Eight implants from each system were included used in the test group (*n* = 24) for experimental laser irradiation under water spray on the upper implant unit, whereas surfaces on the lower unit of the same implants (*n* = 24) were “treated” with water‐spray only (control group A). Four pristine implants from each system had no treatment and were included in control group B (*n* = 12) (Figure 1). All implants were received sterile in the manufacturer's original packaging: OsseoSpeed (OS) TX; Astra Tech Implant System (D, 4.0 mm; L 13.0 mm); TiUnite (TiU) dental implants; Nobel Biocare (D, 4.0 mm; L 13.0 mm), Replace Select TC, RP; Roxolid SLActive (RS), BL, Straumann SLA dental implants (D, 4.0 mm; L 13.0 mm).

**Table 1:** Implant surface information

Implant system	Core material	Surface modification	Surface roughness
Osseospeed	CP Ti grade 4^a^	TiO_2_ particle (25 µm) blasted, Hydrogen Fluoride (HF) acid etched	Moderately rough
TiUnite	CP Ti grade 4^a^	Anodic oxidation process in phosphoric (P) acid electrolyte	Moderately rough
Roxolid SLActive	Ti (85%) Zr (15%) alloy	Large AlO_2_ particle blasted, acid etched (HCl/H_2_SO_4_)	Moderately rough

*Note*: Manufacturer's information.

^a^ ASTM grade. Moderately rough defined as mean surface height (*S*_a_) 1–2 µm. Astra Tech Implant System, OsseoSpeed TX, Dentsply Sirona Implants, Mölndal, Sweden; Nobel Biocare, TiUnite dental implants; Replace Select TC, RP; Nobel Biocare AG, Kloten, Switzerland; Straumann SLA dental implants; Roxolid SLActive, BL, Straumann AG, Basel, Switzerland.

### 2.2 Laser system

The laser system used was a prototype laser (Biolase Inc., Irvine, CA) combining two wavelengths of laser light; one free‐running pulsed 2780 nm Er,Cr:YSGG laser and one 940 nm diode laser operating in continuous wave mode (Table 2). Thus, the laser was operated as a double‐wavelength laser. The laser beam delivery system was an optical fiber, providing a Gaussian beam by a Gold handpiece, equipped with an MZ8 fiber tip at 6 mm length, 800 μm diameter, 8° divergence angle, and 0.14 numerical aperture (NA).

**Table 2:** Properties of the double wavelength laser device (manufacturer's information)

Laser properties	Laser 1	Laser 2
Laser wavelength (nm)	2780	940 ± 10
Mode of operation	Free running pulsed	Continuous wave (cw)
Number of emitters	1	1
Emitter type	Er;Cr:YSGG	InGaAsP, semi‐conductor, diode
Pulse duration	60 μs	
Pulse repetition rate	50 Hz	

### 2.3 Irradiation of the implants

The test treatment was conducted under validated settings based on previous calibrations of double wavelength irradiation (Fahlstedt et al., 2020) (Table 3).

**Table 3:** Output power/energy

Laser/implant system	Output power^a^	Peak power^b^	Pulse energy	Fluence/pulse^c^	Fluence/pulse^d^
	(W)	(W)	(mJ/pulse)	(J/cm^2^)	(J/cm^2^)
Er,Cr:YSGG laser					
OsseoSpeed	1.08	360.0	21.6	8.60	4.30
TiUnite	0.92	306.7	18.4	7.32	3.66
Roxolid SLActive	1.81	603.3	36.2	14.40	7.20
940 nm diode laser				Intensity (W/cm^2^)	Intensity (W/cm^2^)
all implants	3.32	‐	‐	1321.0	660.5

*Note*: water spray volume: 29.2 ml/min.

^a^ Measured value.

^b^ Calculated values from output power.

^c^ Gaussian beam.

^d^ Flat beam.

A Computer Numerical Control (CNC) (Lase‐o‐Matic, Viking; ILSD Sweden AB, Stockholm, Sweden) prototype device was used for rotation of the implants. Furthermore, the fixed laser handpiece in the CNC device had an angle of 90° to the long axis of the implant and a repeatable vertical movement pattern simulated clinical manual debridement. The distance between the fiber tip and implant surface of 1.0 mm was controlled by a calibrated USB microscope (Dino‐Lite/Europe, Naarden, Netherlands), and output power was controlled by a calibrated thermal sensor (FL250A‐BB‐50; Ophir Photonics, Darmstadt, Germany) and universal power meter (Vega Standard, P/N 7Z01560; Ophir Photonics). Before and after each irradiation, output laser power from the fiber tip was recorded. The movement speed was horizontally 3.3 mm/s, and each vertical step of every turn was 0.5 mm. Each implant was vertically divided into two areas covering 63 mm^2^ (4.5 × 14 mm, height × circumference).

In the test group, the upper half part (unit) was irradiated with a double wavelength laser and water spray for 190 s (Fahlstedt et al., 2020). In control group A, the lower unit underwent 190 s of similar laser handpiece/implant movement pattern with water spray from the handpiece, without laser irradiation. The water‐spray temperature was kept between 22.0°C and 24.0°C.

### 2.4 Data assessments

A combination of SEM and a built‐in energy‐dispersive X‐ray spectroscope (EDX) was used for descriptive microstructure evaluation of the implant surfaces and analysis of the chemical composition (elementary semi‐quantitative analysis), respectively (Goldstein et al., 2017). Implants were randomly chosen and carefully mounted horizontally to the sample holder using double‐sided carbon tape, and standardized areas of the implant units were localized and evaluated. As part of the standard procedure before SEM/EDX analysis, RS implants stored in NaCl solution were rinsed with water (Type‐1, 18.2 MΩ), then 15 min sonication in water, rinsed with water, 15 min sonication in isopropyl alcohol, and dried at 120°C for 20 min. For the following surface topography evaluation, a 3D optical profilometer (Interferometer) using a white light laser, was used. Before the interferometry, the implant was mounted with adhesive to the sample holder without further washing or rinsing.

### 2.5 Qualitative surface characterization

**Figure 2:**
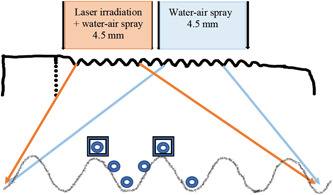
Areas analyzed. For SEM (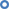) and EDX (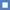), second and third larger threads were localized on upper respective lower unit of each implant: SEM images were taken at six sites: two tops, two flanks, and two valleys, and EDX measurements at two tops per unit. For untreated implants, the middle two threads were measured at nine sites. EDX, energy‐dispersive X‐ray spectroscope; SEM, scanning electron microscopy.

For qualitative SEM surface characterization, a Zeiss GeminiZeiss FEG‐SEM (Sigma with Gemini optics Scanning Electron Microscope; Zeiss Microscopy, Jena, Germany) was used. Due to the thread shape of the implants, images were recorded at the following sites: standardized images of the top, flank, and valley, in two areas of each implant (Figure 2). General images were recorded at magnifications ×200 and ×500. In addition, representative surfaces were recorded at ×5000, ×20,000, and ×60,000 magnification for all implants.

The settings for SEM were Signal A = InLens, EHT = 3.0 kV, and WD = 3.7 mm. Based on the SEM evaluation, the implant unit areas were allocated into two groups: areas with no alteration (no surface alteration group) or areas with an infraction, cracking, melting, or ablation of the surface (surface alteration group).

### 2.6 Chemical composition

The chemical composition of the implant surfaces, analyzed with a build‐in EDX (EDS) equipment (Ultim Max 100 mm^2^ EDS detector; Oxford Instruments, Oxford, UK) was determined by mapping analysis at two standardized areas of thread tops per unit (Figure 2).

AZtec software (Aztec Software Associates, Springfield, NJ, USA) was used for data analysis. The software was set to detect the elements automatically, with obtained data representing the atomic percent. The mapping area was approximately 350 × 100 µm at ×500 magnification and the settings were Signal A = InLens, EHT = 10 kV, and WD = 8.5 mm.

### 2.7 3D quantitative and qualitative surface characterization

**Figure 3:**
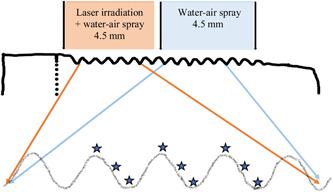
Areas analyzed. For interferometry using a Profilometer (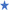), second, third, and fourth larger threads were localized on upper respective lower unit of each treated implant, and nine sites measured: three tops, three flanks, and three valleys for calculations of marginal mean values. For untreated implants, the middle three threads were measured at nine sites.

For the surface topography evaluation, an optical interferometer (smartWLI extended; GBS, Ilmenau, Germany) was used. Each implant unit was analyzed at three different locations: top, flank, and valley, at three different threads (Figure 3). Standardized areas of 350 × 220 µm on each implant's upper and lower unit were localized and analyzed by using ×50 objective and a Gaussian filter with a size of 50 × 50 µm. For qualitative characterization, the surface orientation was evaluated by 3D images and described as isotropic (no orientation) or anisotropic (clear orientation).

For quantitative characterization, the surface variation was described in height, spatial direction, and surface enlargement aspects using surface scan software (Somicronic Instrument, Lyon, France). Three parameters were selected; *S*_a_ describes the average height measured in µm (*S*_a_), *S*_ds_ the density of summits over the measured area measured in 1/µm^2^ (*S*_ds_), and *S*_dr_ the surface enlargement compared with a flat reference area measured in % (*S*_dr_).

### 2.8 Statistical analysis

The sample size was based on calculations from a previous study on double‐wavelength laser irradiation on dental implants (Fahlstedt et al., 2020). Mean values and 95% confidence interval (CI) of the elemental composition in atomic percent at two different sites per unit of test and control materials determined with EDX were calculated and reported for each group.

Linear regression models with robust variance estimates, adjusting for repeated measures per implant unit, were applied to calculate mean values for *S*_a_, *S*_ds_, and *S*_dr_. Since each implant unit had multiple measurements, linear mixed‐effects models were used for testing differences in mean values between the test and control groups. The linear model (assuming a normal distribution and an identity link function) includes a fixed effect part applying a dummy coding for the three sites and the three treatment categories. The implant unit was entered as a random effect adjusting for the correlation between different measures for the same implant. These analyses presented estimated marginal mean values and 95% CI based on the estimated fixed effects and reported for each group. Overall values were presented, and pairwise comparisons when the overall values were significant. Post hoc analyses for multiple comparisons of differences between the test group and control groups were adjusted using Scheffe's method. Results were considered statistically significant for *p* < .05. Stata version 16 (Stata Corp., College Station, TX) was used for all analyses.

## 3 RESULTS

### 3.1 Qualitative surface characterization

**Figure 4:**
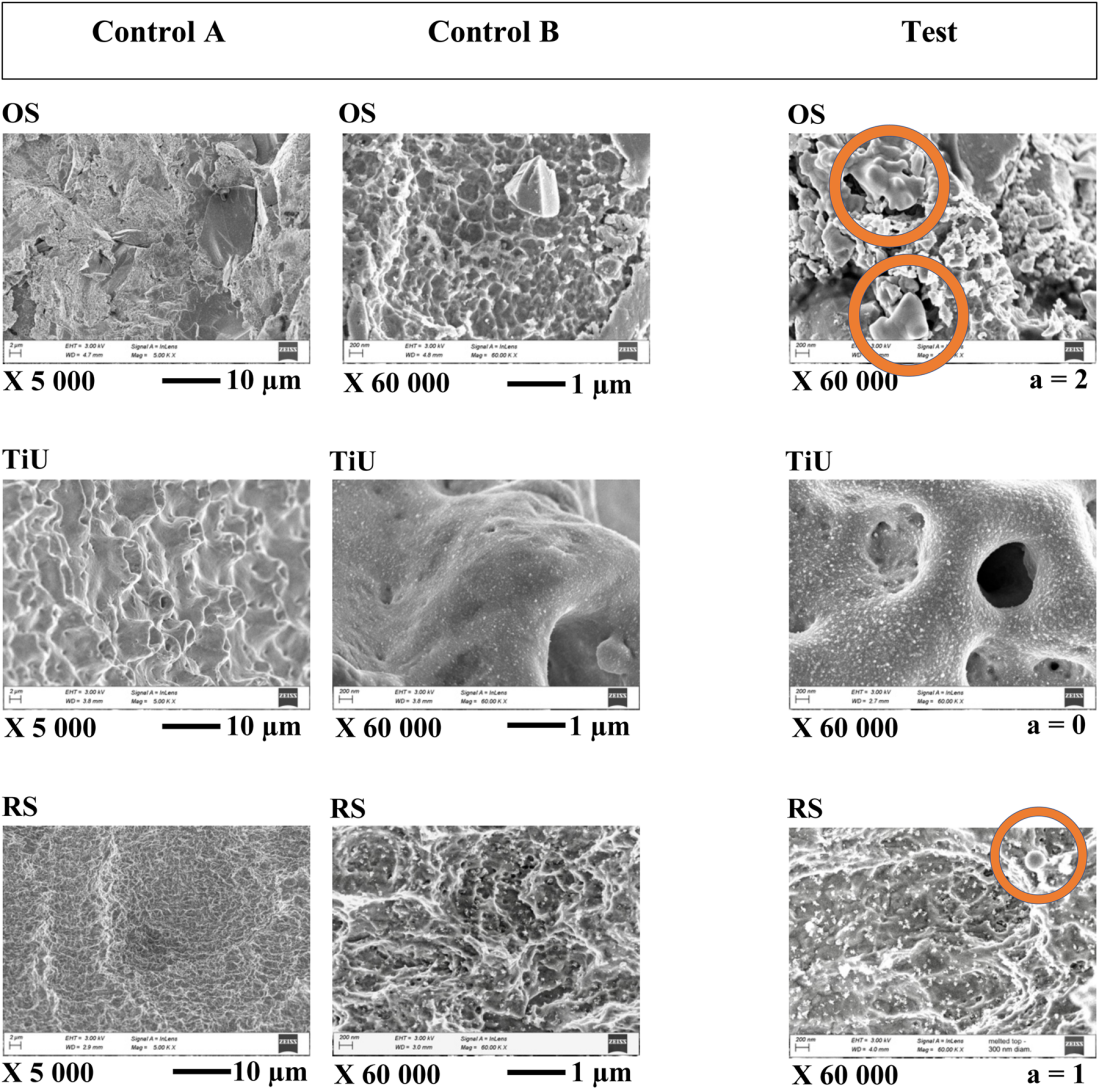
Representative SEM images of implant surface micro‐, nano‐morphology for each implant system. No differences were observed between control A and control B and Test in‐between respective systems, except for two areas (a) with minor (size approx. 1 µm) melted remnants of blasting particles (TiO_2_) for OS and one observation of melted crest top (size approx. 0.2 µm) for RS. Observed signs of alteration, indicated by circles were in the Test group. SEM, scanning electron microscopy.

The surface structure of the different implant systems is depicted in representative SEM images (Figure 4). All implant systems showed an isotropic micromorphology with no precise orientation of structures. The following implant system characteristics were observed: OS: irregular morphology with sharp peaks/valleys and impressions and remnants of sharp‐edged 1–10 μm TiO_2_ blasting particles with nano‐sized structures heterogenically distributed; TiU: smooth morphology with micro‐sized rounded peaks and pits with nano‐sized porosities and structures, homogenously distributed; RS: irregular morphology with micro‐sized irregular peaks/valleys and ridges with nano‐sized porosities and particles, homogenously distributed.

Based on the predefined criteria, 70 OS, 71 RS, and 72 TiU unit areas were included in the No surface alteration group, whereas two OS, none TiU unit, and one RS area were included in the Surface alteration group. Similar nano‐sized structures were observed in all groups of each implant system, while size, amount, and distribution differed among the systems (Figure 4).

### 3.2 Chemical composition

Chemical composition and differences between the groups of each implant system are summarized with overall *p*‐values in Table 4, while specified *p*‐values between groups are presented below. For OS, the concentration of oxygen (O) was significantly higher for the test compared with control B (*p* = .039). For TiU, a significantly higher concentration of carbon (C) was detected in the test compared with control B (*p* = .008), whereas the content of O was significantly lower for control A compared with control B (*p* = .010). For RS, a significantly lower content of titanium (Ti) was observed for test and control A compared with control B (*p* < .001). The content of zirconium (Zr) was significantly lower for the test compared with control B (*p* < .001), and a significantly higher content of O was detected for test compared with both control A (*p* = .021) and control B (*p* < .001).

**Table 4:** Mean values (with 95% CI) for each element (overall *p*‐value for groups tested against each other), in atomic percent of chemical composition of treated and untreated surfaces.

Implant/element	Test group (T)	Control group A	Control group B	Overall *p*‐value
Osseospeed				
Ti	47.1 (44.5–49.7)	47.9 (46.0–49.8)	51.3 (48.6–54.0)	.054
C	3.4 (2.9–3.8)	3.8 (2.7–4.9)	3.3 (2.4–4.2)	.721
O	**49.5 (47.3–51.7)**	48.3 (47.3–49.2)	**45.4 (43.1–47.7)**	**.029** ^**TB**^
F	‐	‐	‐	
TiUnite				
Ti	28.2 (27.2–29.2)	27.8 (26.0–29.6)	28.3 (28.0–28.5)	.893
C	**3.2 (2.6–3.8)**	4.1 (2.1–6.0)	**2.3 (2.1–2.4)**	**.002** ^**TB**^
O	63.0 (61.7–64.4)	**62.7 (61.8–63.6)**	**64.2 (63.8–64.6)**	**.005** ^**AB**^
P	5.5 (5.3–5.7)	5.3 (5.1–5.6)	5.3 (5.1–5.5)	.174
Roxolid SLActive				
Ti	**70.1 (69.0–71.2)**	**70.8 (69.2–72.4)**	**74.8 (74.0–75.6)**	**<0.001** ^**TB,AB**^
C	4.5 (3.9–5.2)	5.0 (4.4–5.6)	4.2 (4.0–4.4)	.058
O	**18.9 (18.0–19.8)**	**16.9 (15.8–18.0)**	**15.4 (14.7–16.0)**	**<0.001** ^**TA,TB**^
Zr	**5.3 (5.1–5.4)**	5.5 (5.2–5.7)	**5.6 (5.6–5.7)**	**<0.001** ^**AC**^
Na	0.7 (0.1–1.4)	1.1 (0.2–2.4)	0.5 (0.1)	.053
Cl	0.4 (0.1–0.9)	0.7 (0.2–1.6)	0.3 (0.4)	.063
N	‐	‐	‐	

*Note*: Capital letters (T, A, and B) in superscript to the overall *p*‐values denote a significant difference (*p* < .05) between the groups. Titanium (Ti), carbon (C), oxygen (O), fluorine (F), phosphorus (P), Zirconium (Zr), sodium (Na), chlorine (Cl), nitrogen (N).

### 3.3 3D qualitative and quantitative surface characterization

Images from white light interferometry for OS, TiU, and RS, revealed an isotropic surface without any structural orientation or observed differences between the groups (test, control A, control B) for each implant system. The TiU surface structure differed along the implant's length and between the orientation of the analyzed area (top, flank, and valley) without observable differences between the groups. Nano‐scale spikes were observed in all groups, with variations related to the orientation of the area analyzed but not between the groups.

The quantitative surface characterization is depicted in Table 5, presenting marginal mean group values with overall p‐values for each parameter, respectively. Specified p‐values between groups are presented in the text below. For OS surfaces, statistically significant lower mean surface height (*S*_a_) and hybrid parameter (*S*_dr_) values were observed for the test compared with control B (*p* = .045, *p* = .042 respectively). There were no statistically significant differences for TiU, between the groups for any parameter, while the RS hybrid parameter (*S*_dr_) parameter was significantly higher for control A compared with control B (*p* = .006).

**Table 5:** Mean values (with 95% confidence intervals) for each parameter (*S*_a_, *S*_ds_, *S*_dr_) for all groups (overall *p*‐value for treatments tested against each other).

Implant/parameter	Test group: laser/water	Ctr. group A: water	Ctr. group B: no treatment	Overall *p*‐value
Osseospeed				
*S*_a_ (µm)	**1.98 (1.86–2.10)**	2.09 (1.99–2.17)	**2.20 (2.15–2.24)**	.**039** ^**TB**^
*S*_ds_/µm^2^	0.18 (0.18–0.18)	0.18 (0.17–0.18)	0.19 (0.18–0.20)	.126
*S*_dr_ (%)	**119.98 (91.32–148.63)**	133.66 (107.37–159.95)	**176.76 (165.03–188.49)**	**.041** ^**TB**^
TiUnite				
*S*_a_ (µm)	3.78 (3.25–4.33)	3.82 (3.25–4.39)	3.74 (2.96–4.52)	.985
*S*_ds_/µm^2^	0.22 (0.22–0.23)	0.22 (0.21–0.23)	0.21 (0.20–0.23)	.474
*S*_dr_ (%)	694.85 (575.97–813.72)	698.74 (574.62–822.87)	659.96 (499.88–820.04)	.925
Roxolid SLActive				
*S*_a_ (µm)	2.07 (1.95–2.18)	2.19 (2.09–2.29)	2.01(1.82–2.20)	.094
*S*_ds_/µm^2^	0.16 (0.16–0.17)	0.16 (0.16–0.17)	0.16 (0.16–0.16)	.535
*S*_dr_ (%)	78.43 (73.58–81.95)	**91.09 (80.69–101.50)**	**69.03 (63.31–74.75)**	**.003** ^**AB**^

*Note*: Capital letters (T, A, and B) in superscript to the overall *p*‐values denote a significant difference (*p* < .05) between the groups, based on Scheffe's method. Marginal mean values calculated based on values from each measured site (top, flank, valley).

## 4 DISCUSSION

The results demonstrate minor changes in micro‐morphology for irradiated OS and RS implant surfaces compared with control implants. Significant differences in chemical composition were observed between irradiated and water‐sprayed surfaces within all implant systems compared with untreated control. Moreover, there were significant differences in surface roughness between laser‐irradiated OS surfaces and untreated control, with the highest roughness for the control group.

### 4.1 Qualitative surface characterization by SEM

The first signs of alteration in the micromorphology were observed from ×5000 magnification and higher, and the first nano‐sized particles were observed at ×20,000 to ×60,000 magnification. Previous studies analyzing laser‐treated titanium implants/discs have precluded surface evaluation on a nano‐sized level due to rather low ×50 to ×5000 magnification (Ercan et al., 2015; Larsen et al., 2017), and recently, ×10,000 to ×12,000 magnification (Alagl et al., 2019; S. H. Park et al., 2020).

The observation that no implant system showed in‐between group differences in isotropic surface orientation following Er,Cr:YSGG laser irradiation corresponds with previous studies where different output energies were tested (Chegini et al., 2020; Ercan et al., 2014). However, less than 3% of the OS's areas showed single altered TiO‐particles (blasting particles) in the microstructure following laser irradiation, with no alteration of the core titanium. One single droplet‐like (0.2 μm) melted part of a blasting particle (30 µm) was observed at one area of RS. The findings may be related to a temporary insufficient amount of water spray or decreased distance from the implant surface to the fiber tip. Both factors increase photons' fluence (energy/area) reaching the implant surface. Consistently, high thermal energies in TiO_2_‐particles (OS) and thin Ti‐Zr alloy (RS) peaks may result in melting. These findings are congruent with previous studies, where fluences exceeding the breakdown or melting point of the titanium surface have resulted in similar alterations increasing in area and distribution depending on applied energies from pulsed erbium‐lasers (Chegini et al., 2020; Fahlstedt et al., 2020; Taniguchi et al., 2013).

### 4.2 Chemical composition by EDX

Besides concentrations of Ti, O, and C elements expected to be found in the present study are P for TiU from the acid electrolyte, F for RS from HF acid etching, core alloy (Ti/Zr), N from cleaning and Na and Cl from storage (Wennerberg et al., 2015). The results confirm these system‐related differences, even though superficially positioned content of F and N were not detectable in any group for RS, directing EDX's deep detection depth. For analyses of the uppermost nano‐layer of the titanium surface, X‐ray photoelectron spectroscopy (XPS) with a detection depth of approximately 5–10 nm is recommended. However, Er,Cr:YSGG and diode 940 nm laser energies have high absorption and thermal conductivity in titanium and TiO_2_, even in the bulk material under the uppermost layer. Moreover, the potential for parallel specimen analyses by EDX and SEM in the same setting probably reduces the interfering impact and the contamination of the implant surface.

The HF acid etching produces nanostructures, shown to offer the improved ability of the titanium surface to osseointegrate (Lamolle et al., 2009). Despite not detecting F content, the nanostructures evaluated by SEM, were unaffected in all groups, regardless of treatment. Coinciding, a similar lack of nanostructure alterations following Er:Cr:YSGG laser irradiation was found in an in vitro study following the decontamination of failed SLA implants (Secgin‐Atar et al., 2021). These observations indicate that the nano‐sized morphology is preserved potentially maintaining the ability of re‐osseointegration.

In a previous in vitro study, Er:YAG laser irradiation under water spray of SLA discs resulted in an increased superficial layer of TiO_2_ (Scarano et al., 2020). Ti is highly reactive, including elements with a low atomic number like O. While the exposition of O may lead to the oxidation of Ti (TiO_2_), a higher concentration of O leads theoretically to lower relative concentrations of other elements on the titanium surface (Wennerberg et al., 2015). In contrast to OS and RS, the TiU's O concentration was significantly lower for control A surfaces than for control B, while the carbon (C) concentration was higher. The results indicate an impact of both laser irradiation and water spray on O and Ti concentrations for all implant surfaces, with a clinically unknown effect on the ability to reosseointegrate. However, it has been shown that an increased oxide layer may promote a hydrophilic surface with increased free energy and wettability, both factors promoting osseointegration (Kilpadi & Lemons, 1994).

Comparing test surfaces with control A, elemental concentrations were not significantly different except for RS, where the O concentration was higher for the test. The results indicate an overall minor influence of laser irradiation on the chemical composition and coincidence with an in vitro study on Grade 2 Ti‐alloy (Yao et al., 2020). Minor differences in the chemical composition, analyzed by XPS, were found following Er,Cr:YSGG irradiation.

### 4.3 3D qualitative and quantitative surface characterization by light interferometry

The validated laser irradiation did not influence the isotropic structural orientation or the micro‐and nano‐sized structure, coincident with the observations of the SEM images. Observed nano‐scaled particles differed in orientation and concentration depending on the shape of the threads but were similar between the treatment groups of each implant system. The findings correspond with observations following Er,Cr:YSGG laser irradiation of micro‐textured titanium discs (Ercan et al., 2015). No qualitative surface alterations were detected when using different power settings.

Previous in vitro studies have shown a relationship between laser fluence (energy/area) and the effects on titanium disc surface topography. However, the results were inconsistent showing both an increase (J. H. Park et al., 2012) and a decrease (Ercan et al., 2014) in surface roughness *R*‐values (2D profile parameters) following irradiation with increasing fluence. The evaluation methods and type of titanium surface analyzed differed among studies and the findings are not directly comparable.

The surface roughness values (*S*_a_, *S*_dr_) for OS following test treatment were lower than for control B, indicating that the laser irradiation smoothened the surface. In contrast, laser irradiation did not significantly influence any roughness parameter for TiU or RS surfaces. This indicates that, compared to TiU and RS, the chosen fluence used for OS is probably closer to surface breakdown level and an optimized setting for effective decontamination of biofilm/calcifications.

The RS's hybrid parameter (*S*_dr_) was significantly higher in control A than in control B, demonstrating more influences on the topography from water spray than the combination of laser irradiation and water spray. This finding is delicate to explain. A possible variation in the manufacturing process or physical forces from the handling of the implants at the laboratory tests may be a contributing factor (Dias et al., 2020). Moderately rough surface with *S*_a_ values of approximately 1.0–2.0 μm and *S*_dr_ of approximately 50% is generally accepted as promoting the strongest bone‐stimulating responses (Wennerberg and Albrektsson, 2010). Following control A and B treatment, the implants presented rough (>2 µm) surfaces, while test surfaces were moderately rough. These surface alterations may have bioclinical implications.

The authors acknowledge some limitations of the study. First, the chemical compound detected by the EDX corresponds to a mean of elements in the bulk of the material on the surface. In addition, with a detection depth of 1 μm, the technique is not entirely surface‐specific, missing the most superficial elements. Second, only three titanium dental implant surfaces were examined, limiting the general applicability of the observations. Included implants were selected based on their frequent use worldwide and representing different surface modifications. Third, the standardized in vitro setup is not obtainable in clinical settings, given the design of today's commercially available fiber tips.

Within the study's limitations, it can be concluded that for each implant system, the validated double wavelength laser irradiation did not change the micro‐, and nano‐sized structures. Double wavelength laser irradiation influenced the chemical composition primarily of oxygen for all implant systems and smoothened the surface of OS implants. The impact of laser irradiation on the ability to re‐osseointegrate remains unknown, the ambiguous influence of the surface chemical compound and surface roughness needs to be investigated in further studies. Further, evaluating the double wavelength laser's debridement and decontamination efficacy is motivated.

## AUTHOR CONTRIBUTIONS

All authors have made substantial contributions to conception and design of the study. Peter Fahlstedt and Ann Wennerberg collected the data and Stein A. Lie analyzed the data. Peter Fahlstedt, Ann Wennerberg, Dagmar F. Bunæs, Stein A. Lie, and Knut N. Leknes have been involved in data interpretation, drafting the manuscript, and revising it critically and have given final approval of publishing.

## CONFLICTS OF INTEREST

The authors declare no conflicts of interest.

## Data Availability

The data that support the findings of this study are available from the corresponding author upon reasonable request.
